# Can second language metaphorical competence be taught through instructional intervention? A meta-analysis

**DOI:** 10.3389/fpsyg.2022.1065803

**Published:** 2022-11-30

**Authors:** Xiaoyong Zhou, Muhammad Younas, Abdulfattah Omar, Lu Guan

**Affiliations:** ^1^School of Foreign Languages, East China Normal University, Shanghai, China; ^2^School of Education, Soochow University, Suzhou, China; ^3^Department of English College of Science & Humanities, Prince Sattam Bin Abdulaziz University, Al-Kharj, Saudi Arabia; ^4^Shanghai Minhang No.3 Middle School, Shanghai, China

**Keywords:** metaphorical teaching, competence, second language, instructional intervention, meta-analysis

## Abstract

**Background:**

For a long time, the traditional view regarded metaphor as merely a rhetorical device that served to enrich linguistic expression. With the continuous development of cognitive linguistics, foreign language educators began to realize the vital role of metaphor in foreign language education.

**Objectives:**

This study looked at how well pedagogical interventions improve metaphorical competence by looking at how well teachers teach metaphors.

**Methods:**

After a rigorous literature search and selection process from the Chinese and English databases, 13 Chinese and 7 international studies with 51 effect sizes were included in this meta-analysis. With the help of the meta-analysis 3.0 software, the literature and heterogeneity tests were performed to ensure that the meta-analysis results were as accurate and valid as possible.

**Results:**

The effect size tests revealed that the metaphorical instructional intervention was significantly effective in general and produced a large effect size (d = 0.888) on improving learners' metaphorical abilities. Meta-regression analyses were also conducted to examine how other factors might change the effects of the interventions.

**Findings:**

Research has shown that instructional interventions that combine prolonged input of metaphorical concepts with reinforcement of metaphorical practice can help second language learners develop metaphorical competence. Teaching puts more pressure on teachers and the learning environment, and the results of this study could help teachers teach metaphors in the future.

## Introduction

Metaphor, a cognitive tool for cross-domain mapping between different conceptual domains, is widely recognized as the primary and fundamental means of human existence. Second language learners talk using the target language's vocabulary and structure; they are sometimes accustomed to thinking and behaving in terms of their native language's conceptual system, resulting in a large number of “unnatural” expressions (i.e., Charteris-Black, 2002; Jarvis and Pavlenko, 2008; Littlemore et al., 2018). Theoretical and empirical studies illustrated that the competence of understanding and producing metaphors in the target language is essential for language learning from vocabulary learning, reading comprehension, and writing production (Kathpalia and Carmel, 2011; Wang and Cheng, 2016; Hoang and Boers, 2018). Relationships between metaphorical competence and language proficiency, cognitive style, cognitive ability, gender, age, language transfer, and creativity were under consideration (e.g., Chiappe and Chiappe, 2007; Kenett et al., 2018; Fattahi and Nushi, 2021).

The ability to generate and understand metaphors in second language learning can help us understand, expand ideas, and clarify old problems and concepts, as metaphors are common in our daily lives (Abdul-Zahra, 2015). Researchers demanded that they interpret and write target expressions literally and metaphorically into a short passage (Card, 2015; Afzaal and Xiangyi, 2020; Haberman et al., 2020). It is claimed that metaphor can be found in almost every third English sentence (Arif and Abdullah, 2017). Azuma (2004) and Kanglong and Afzaal (2020) designed various metaphorical ability tests, including the receptive and productive tests. Researchers' perspectives on the components of metaphorical competence differ due to differences in research focus and objectives.

Several examples illustrate that the use of metaphor is pervasive in everyday language, which reflects how we think and act. (Karadag, 2015). With the deep influence of both traditional and cognitive views, metaphor is not only seen as a rhetorical device in language but, more importantly, a way for people to recognize new things related to human cognition (Haji, 2011). Norafkan (2013) introduced the concept of metaphorical competence into second language acquisition and foreign language teaching. It is believed that metaphorical competence involves metaphorical awareness, comprehension, and expression strategies, and it is advocated that all second language learners need to develop some skills related to metaphor (Pavlenko, 2009; Li, 2010). Tafazoli and Piri (2018) stressed that metaphorical competence should include the ability to understand and learn metaphors passively as well as the ability to use metaphors creatively. Soureshjani and Safikhani (2012) conducted an empirical study and reported that the receptive metaphorical competence of Chinese learners could correlate with their reading proficiency. Toyokura (2016) examined four aspects of metaphorical skills: metaphorical fluency, metaphorical flexibility, metaphorical originality, and metaphorical elaboration, and showed that English proficiency could determine metaphorical creativity to some degree. Studies propose that metaphorical competence in an EFL context should concern learners' ability to recognize metaphorical expressions in listening and reading materials, apply appropriate metaphorical terms in oral or written form, and comprehend concepts behind metaphorical expressions (Hussey and Katz, 2006; Galantomos, 2019; Liu and Hsieh, 2020).

Metaphor learning is relatively flexible, requiring second language learners to change their view of traditional language learning focused on grammar and language forms. The metaphor may help L2 writing instructors analyze student needs, boost metacognition, and delve into aspiring economics instructors' personal and emotional metaphors about the roles they would play as teachers (Mellado et al., 2021; Yang and Peng, 2021). Studies investigated whether embodiment or abstraction is used in processing Chinese verbal metaphors and used event-related potential testing to compare the electrophysiological processing of scientific metaphors in Chinese and English to understand better the differences between the processes of perceiving figurative language (Alfadda et al., 2022; Hu et al., 2022; Li et al., 2022; Tang et al., 2022). By putting metaphorical ideas into practice and using them in teaching, students can see and experience new metaphorical ways of thinking, which helps them form metaphorical ways of thinking habits.

### Study statement

The importance of developing learners' metaphorical competence is self-evident, given the crucial role of metaphorical competence in many aspects of language teaching and learning. However, in recent decades, there has been less discussion about the feasibility and impact of a pedagogical intervention on the development of metaphorical competence. The investigation adds to the understanding of the L2 learners' metaphorical competence by improving instructors' metaphor knowledge and increasing metaphor awareness in L2 writing, teaching, and learning (Lu, 2021). The previous study explored hemispheric differences in the processing of metaphors by introducing scientific metaphors as new metaphors and providing orientation mapping from the particular and known domains to the abstract and unfamiliar domains (Huang et al., 2022). However, many attempts have been made to further define the idea of metaphorical competence due to the differences in research focus and objectives; researchers have different views on the components of metaphorical competence to fill the research gap. This study collects, sorts out, synthesizes, and analyzes the results of multiple similar studies in the existing empirical literature and then carries out the qualitative and quantitative analysis in a more overall and systematic way using a meta-analysis approach. In recent years, meta-analysis has gradually emerged in second-language research abroad, but this method needs to be better known in the L2 research field in China. So, this research tries to improve the way metaphors are studied by using meta-analysis, which is one of the new things about this research.

## Literature review

The study highlighted the learner's ability to use metaphor as a core ability in second language learning. The study tried to analyze Bachman's widely used model of communicative language competence (Littlemore and Low, 2006). The findings revealed that metaphorical competence was highly relevant to grammatical, textual, illocutionary, sociolinguistic, and strategic competence. Chen (2019) explores how metaphorical competence is related to language proficiency. A study was done to compare the high-level and low-level groups. The results showed that the high-level group gave more metaphorical answers on the sentence cloze test. This meant that high-level learners were better at using metaphors than low-level learners (Mehdipoor et al., 2021).

Similarly, Aleshtar and Dowlatabadi (2014) found a positive relationship between scores on OPT and metaphorical competence among Iranian EFL students. The study investigated the effect of proficiency level on metaphorical competence among Greek learners and drew similar conclusions on the positive relationship of proficiency level with previous literature (Galantomos, 2019). By identifying and quantifying learners' metaphorical expressions, they concluded that the density of metaphorically used words and phrases increased systematically by the year of level and that learners' performance in writing was correlated to their use of metaphorical expressions (Hoang and Boers, 2018). Fattahi and Nushi (2021) recently concluded that upper-intermediate students used metaphors in their writing better than intermediate students.

In China, the discussion on this topic was widespread and pointed out that the relationship is not static but dynamic (Younas and Qingyu, 2021). With increased English proficiency, metaphorical competence significantly improves. This influence has a decreasing trend and further explains that learners lack the awareness to construct an L2 metaphorical system because the learning environment does not pay much attention to developing metaphorical competence (Xiaofang, 2021). An empirical study reported that the receptive metaphorical competence of Chinese learners could correlate with their reading proficiency, and those good at reading showed a high level of metaphorical competence. Chinese learners were still at a low or middle level in metaphorical competence (Zhao et al., 2014). The study examined four aspects of metaphorical competence: metaphorical fluency, metaphorical flexibility, metaphorical originality, and metaphorical elaboration, and showed that English proficiency could determine metaphorical creativity to some degree (Wang and Cheng, 2016).

The case study in China verified that cognitive style had a motivating function for metaphorical competence. Style learners were more sensitive to conceptual metaphors, and analytic style learners performed better in metaphorical comprehension (Wang and Hao, 2013). The study explores metaphorical development from the perspective of cognitive styles, and findings showed that holistic learners performed better with conceptual metaphor instruction. In contrast, instruction with metaphorical mappings is more suitable for analytic learners (Chen et al., 2014). An empirical study (Hashemian, 2018) examines the differences in how people with different learning styles understand metaphors and how affective factors might be related to metaphorical competence.

Metaphorical competence has been shown to play a prominent role in SLA. Theoretical and empirical studies demonstrate that metaphorical competence is closely related to language learning. Charteris-Black (2002) designed their research based on the Boers' analysis, added high- and low-language-level groups, and examined the effect of metaphorical awareness on word form and meaning retention. Interactive and cultural characteristics of the metaphorical cognitive mechanism indicate that the cultivation of metaphorical competence should be crucial for foreign language teaching (Younas and Noor, 2021). They believed that metaphor could contribute to cultural comprehension and the actual use of language. Education of the target language can be achieved through teaching metaphors.

Chen et al. (2014) pointed out that schema mapping of metaphor was a scaffold for learners to deal with discourse and understand the target domain. The cognitive process of metaphor in discourse comprehension has pedagogical value and suggests teaching the target language's culture through metaphor. Empirical studies of English learners in China (Kathpalia and Carmel, 2011; Zhao et al., 2014) found a strong link between learners' receptive metaphorical competence and reading skills. This was done by encouraging people to think about and talk about the world in metaphors.

## Research methods

After clarifying the Research Topic and proposing specific research questions, concrete meta-analysis procedures were applied, as meta-analysis is a method of research integration that involves extracting data analysis results from previous empirical studies. Study coding and effect size analysis followed strict guidelines, including literature search, inclusion and exclusion, study coding, and effect size analysis (Sánchez-Meca and Marín-Martínez, 2010).

A meta-analysis is a statistical tool for summarizing and synthesizing data from several investigations (Koizumi and Tomita, 2019). Ortega and Norris (2006) mentioned that meta-analysis is a method of research integration that involves extracting data analysis results from previous empirical studies for secondary analysis and processing. Several studies are combined; this method may aid in consolidating commonalities and explaining contradictory results (Chong and Plonsky, 2021). It is difficult to evaluate if particular factors aid language learning based merely on statistically significant data from a single study, as different studies may produce contradictory results. A meta-analysis helps synthesize study findings to see whether certain aspects are successful.

### Literature search process

Literature at home and abroad from 2006 to 2020 was searched to find all eligible studies related to the research question as much as possible and ensure that the research results will not be biased due to the omission of specific studies. Literature was mainly searched in three crucial academic databases: CNKI, WAN FANG, and WEI PU. Literature databases include were not limited to Web of Science, Scopus, JSTOR, ScienceDirect, Education Resource Information Center (ERIC), Google Scholar, Academic Research Library (Pro-Quest), and Wiley Online Library. Keywords included 隐喻能力, 概念流利, and corresponding English keywords had metaphorical competence, conceptual metaphor, and conceptual fluency.

### Inclusion and exclusion criteria

By using these Chinese keywords, “隐喻能力” or “概念流利,” 644 documents were searched. Qualitative analysis and reviews were excluded after further reading abstracts and full texts. The meta-analysis looked at 13 pieces of research, leaving out duplicate data and unqualified research.

**Figure 1:**
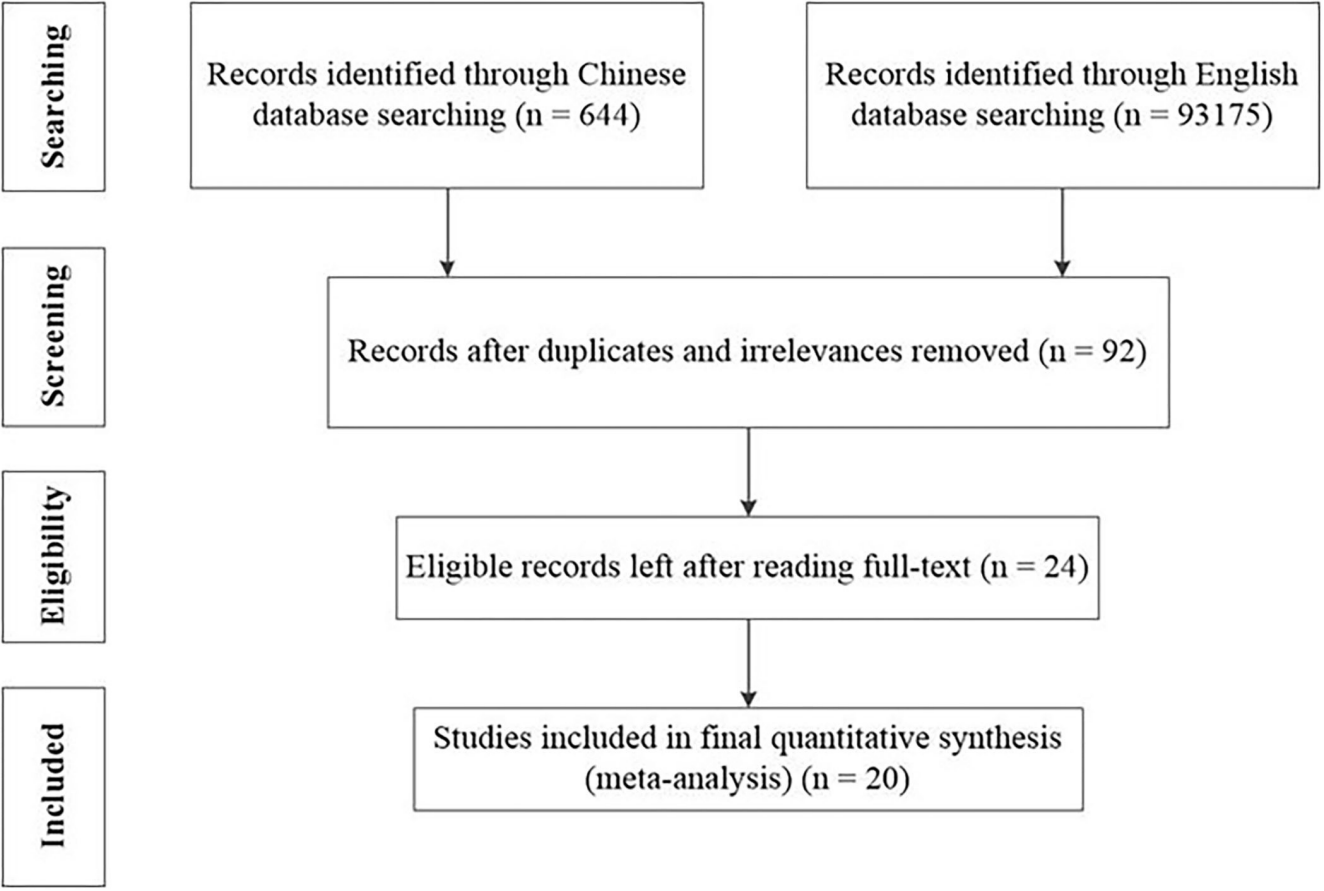
Chart of the study selection process.

For English databases, the search keywords (metaphorical ability, conceptual fluency, and conceptual metaphor) were used, and a total of 93,175 documents were retrieved. To facilitate the screening process, we narrowed the research field to include pertinent subfields, including language acquisition, linguistics, education, pedagogy, and psychology. Reading the titles and abstracts of studies, we were able to narrow our search down to 77, which examined the impact of educational intervention on metaphorical ability after discarding duplicates and irrelevant literature. Seven pieces of international literature were included in this meta-analysis which is shown in Figure 1.

### Coding of studies

Coding for studies allows researchers to clarify the conditions under which different research results were derived. Features served as moderators of effect size by examining and explaining how effect size varies with research characteristics. Coding in the meta-analysis focused on capturing the most likely relevant factors in the change of effect size (Pigott and Polanin, 2020). Features of the study are shown in Table 1.

**Table 1:** Study characteristics.

**Study name**	**Sample size**	**Region**	**Target language**	**Type of MC**	**Intervention** **length**	**Test form**	**Control** **group**	**Source**
Lang and Yi (2019)	30	China	Chinese	Identification, interpretation, production	16w	Test score	With	Journal
Qing (2014)	100	China	English	General	16w	Test score	With	Journal
Dandan (2019)	90	China	English	General	16w	Test score	With	Journal
Yuanyuan (2020)	81	China	English	General	13w	Test score	With	Journal
Ting (2020)	82	China	English	Production	7w	Metaphor density	With	Journal
Yuanlian (2012)	80	China	English	Production, comprehension	Immediate	Test score	Without	Journal
Ming (2009)	66	China	English	Identification comprehension Interpretation	16w	Metaphor density	With	Journal
Jinfang (2015)	50	China	English	General	6w	Test score	Without	Journal
Liang (2013)	66	China	English	Identification interpretation production	16w	Test score	With	Journal
Shan (2016)	60	China	English	Identification, comprehension production	4w	Test score	With	Thesis
Di (2013)	40	China	English	identification interpretation, comprehension production	14w	Test score	With	Thesis
Qinghua (2006)	32	China	English	production, identification, comprehension	24w	Test score	With	Thesis
Wang and Cheng (2016)	91	China	English	Identification	2w	Test score	With	Journal
Chen and Lai (2015)	68	China	English	Identification	2w	Test score	Without	Journal
Hashemian (2006)	139	INT	English	Identification, comprehension production	–	Test score	With	Journal
Ashraf and Majeed (2011)	60	INT	English	General	14w	Test score	With	Journal
Abdul-Zahra (2015)	40	INT	English	Comprehension	–	Test score	With	Journal
Shirazi and Talebinezhad (2013)	20	INT	English	Production	4 sessions	Metaphor density	Without	Journal
Norafkan (2013)	53	INT	English	Production	8w	Metaphor density	With	Thesis
Toyokura (2016)	66	INT	English	Interpretation	12w	Test score	Without	Journal

### Computation of effect size

All included studies reported the standard deviation and mean value of the experimental group and the control group or the difference between the same group of subjects before and after the intervention. The study used Cohen's d as the statistical indicator of effect size. d is a commonly used effect size to calculate the difference between groups, which is calculated by combining the standard deviation of the mean difference. The meta-analysis will convert other statistics into d values using Comprehensive Meta-Analysis V3.0 to unify effect size. The same article reported on several aspects of metaphorical competence; each study and task in this literature will be treated as a separate study. Effect sizes are calculated and labeled separately, and the calculation formula for *d* is as follows:


(1) $$\begin{array}{lll} d & = & \frac{\bar{X_{1}}\text{-}\bar{X_{2}}}{SD_{\text{wi}thin}} \end{array}$$

(2) $$\begin{array}{lll} SD_{within} & = & \sqrt{\frac{\left(n_{1}-1\right)SD_{1}^{2}+\left(n_{2}-1\right)SD_{2}^{2}}{n_{1}+n_{2}-2}}, \end{array}$$

where $\bar{X1}$ and $\bar{X2}$ are the sample means of the two groups, respectively, and *SDwithin* represent the within-group standard deviation.

## Results

### Heterogeneity test

The heterogeneity test was a crucial step in synthesizing the effect sizes of individual studies into the overall effect size and is essentially a test of whether the studies belong to the same distribution. Heterogeneity analysis is commonly evaluated using the Q test and the I^2^ test. The Q-test is based on the total variance, assuming that the effect sizes come from the chi-square distribution, and if p < 0.05, the study is heterogeneous. The I^2^ describes the percentage of total variation due to individual studies and not a sampling error, with I^2^ = 25% indicating low heterogeneity, I^2^ = 50% indicating moderate heterogeneity, and I^2^ = 75% indicating high heterogeneity.

According to Table 2, the Q value of the present study equals 557.286, and *P* = 0.000 < 0.05. The Q values reached the significance level. In addition, the I^2^ value was 91.028% > 75%, indicating high heterogeneity, which further justifies the choice of the random effect model.

**Table 2:** Results of the homogeneity test.

	**N**	**Q**	**Df**	**P**	***I^2^*(%)**	**Tau^2^**
Intervention effect	51	557.286	50	0.000	91.028	0.629

### Assessment of publication bias

**Figure 2:**
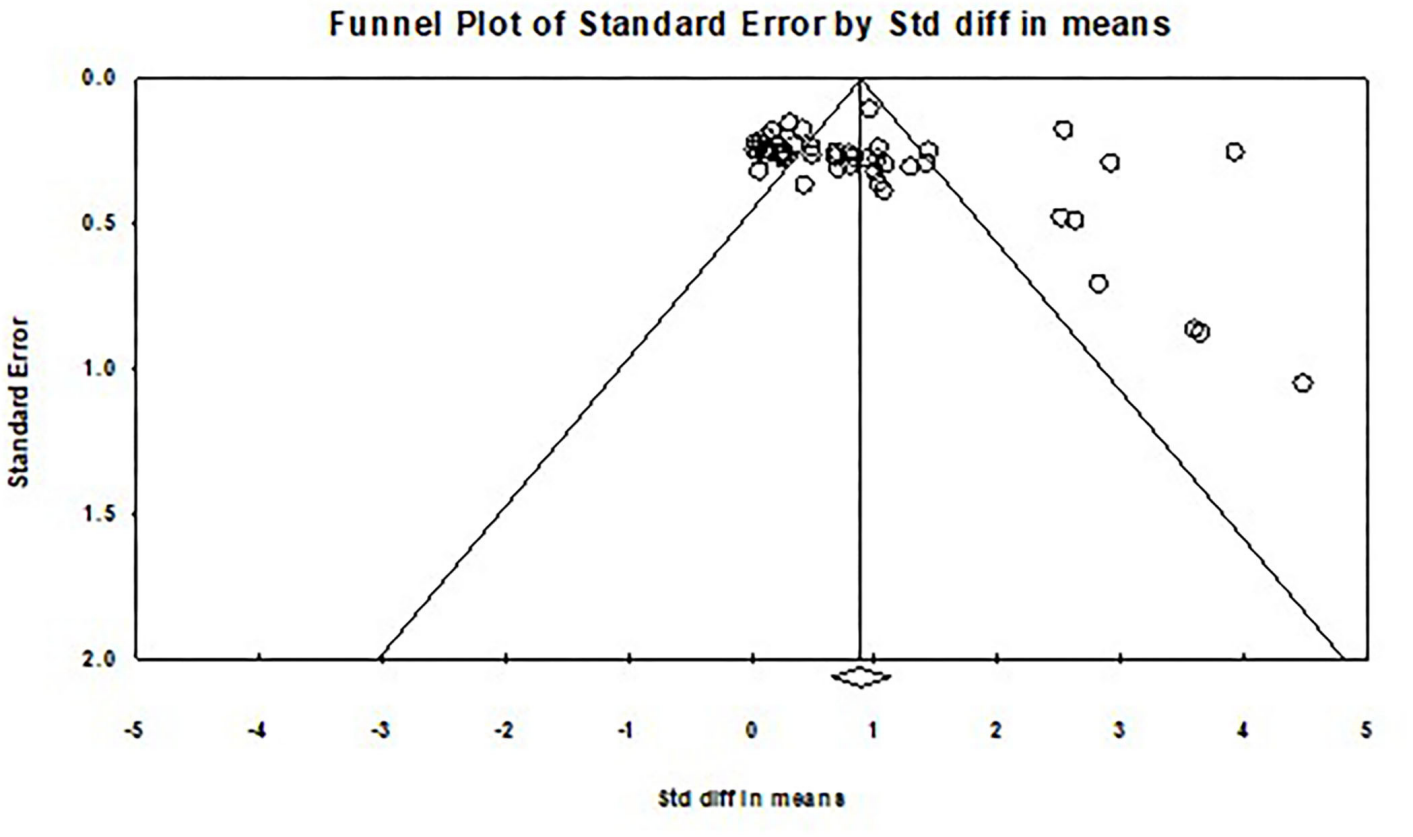
Funnel plot of the overall effect of instruction on metaphorical competence.

By observing the funnel plot shown in Figure 2, most of the independent samples were distributed at the top of the funnel plot and located on both sides of the total effect size. This distribution feature suggests that studies may not have a significant publication bias. While considering those deviating points on the right side, the conclusion should be further checked.

The funnel plot can only initially observe publication bias from a subjective qualitative point of view. Quantitative methods such as Rosenthal's Classic Fail-Safe N and Egger's regression are needed to provide factual data about the degree of publication bias. In the present meta-analysis, the Nfs is 6,382, much larger than 5k+10 (255). It indicates that there is no significant publication bias. At least 6,382 more studies are needed to overturn the results of this study. If linear regression yields an intercept close to 0 and is not significant, then the risk of publication bias is low.

The study used the random effect model to synthesize the overall effect size of explicit instruction on fostering L2 metaphorical competence. Independent studies were included in the meta-analysis, involving a sample size of 851. Table 3, the test results showed that the summary effect was d = 0.88 (*p* < 0.001). According to the effect size evaluation criteria, the immediate effect obtained from the study was more significant than 0.8, indicating that the overall impact of the intervention reached a significantly high level. The 95% confidence interval of the immediate effect size of the intervention was 0.654, 1.123, which does not contain 0. The 95% CI of the effect sizes were all >0, and the horizontal line in the forest plot does not intersect the null vertical line. It is to the right of the invalid vertical line and suggests that the effect can be considered as not occurring by chance. The summary effect supports the claim that the current instructional intervention improves learners' metaphorical competence.

**Table 3:** Summary effect of the intervention on metaphorical competence.

**Model**	**Effect size and 95% confidence interval**					
	**Number studies**	**Point estimate**	**Standard error**	**Variance**	**Lower limit**	**Upper limit**
Fixed	51	0.743	0.035	0.001	0.676	0.811
Random	51	0.888	0.120	0.014	0.654	1.123

**Figure 3:**
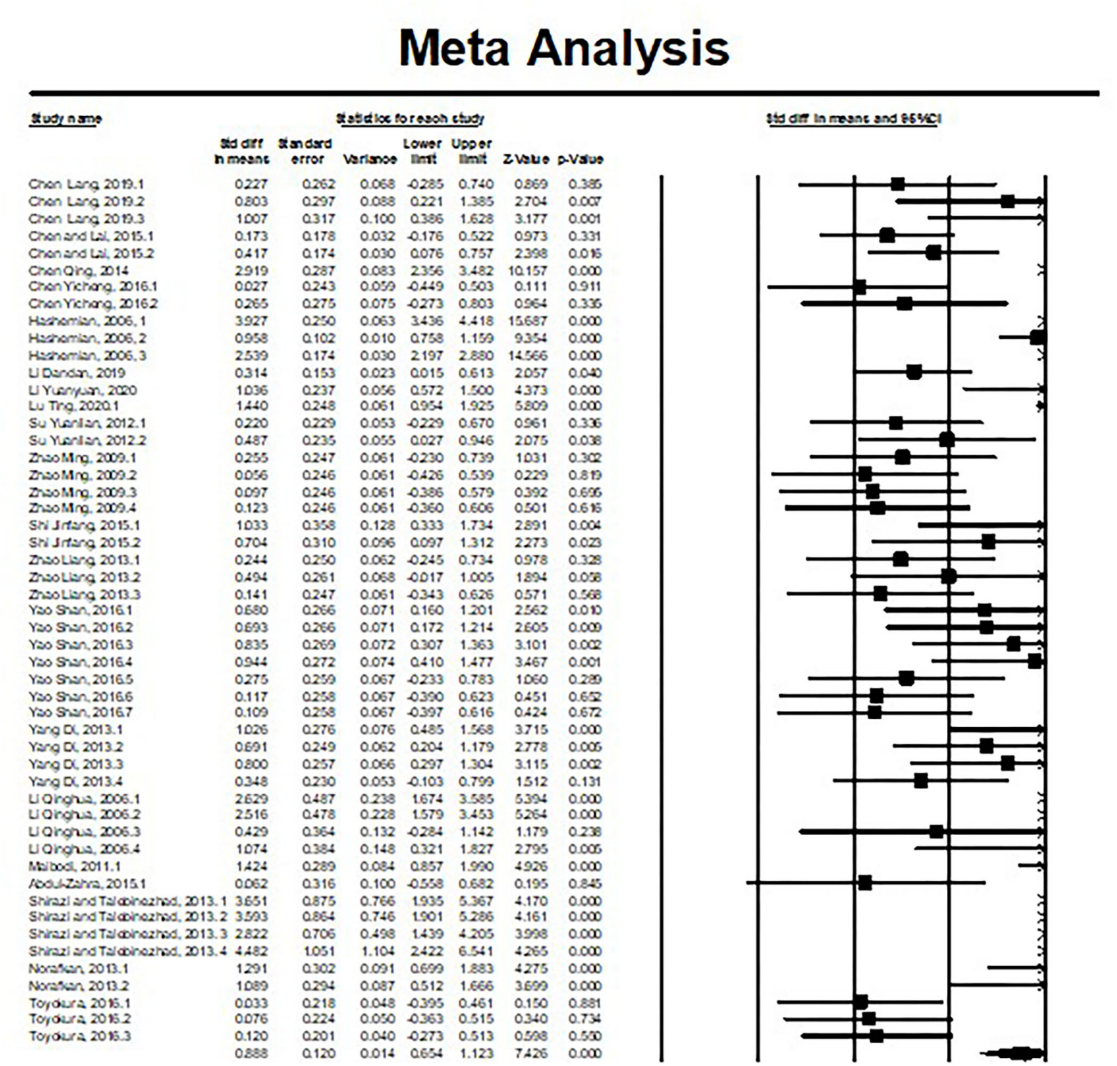
Forest plot of the overall effect of instruction on metaphorical competence.

Moreover, the effect size and confidence interval found in the individual studies are presented in Figure 3.

### Effects of moderators

The heterogeneity between individual studies justifies further analysis of moderator factors. A subgroup analysis will be conducted to determine whether the variables of type of metaphorical ability, region, experimental design, and source of literature moderated the effect of improving learners' second language metaphorical ability by instruction.

### Effects of metaphorical competence type

Through a review of the literature included in the analysis, researchers may have different approaches to evaluate the metaphorical ability of second language learners. The discussion of metaphorical competence in the included literature can be concluded into five types: the competence of metaphorical identification, metaphorical interpretation, metaphorical comprehension, metaphorical production, and general metaphorical competence. Subgroup analysis of the kinds of metaphorical competence indicated that teaching interventions could generate varying degrees of effect on different types of L2 metaphorical competence (Q_B_ = 24.070, df = 4, *P* = 0.000 < 0.01). The specific data in Table 4 shows that teaching interventions have a significant impact on metaphorical comprehension (d = 0.762), production (d = 1.286), and general metaphorical competence (d = 1.425). The average effect of an instructional intervention on metaphorical identification is at a medium level (d = 0.448). A small effect (d = 0.295) was shown in the improvement of metaphorical interpretation. Moreover, there is significant heterogeneity in the effect sizes for three types of metaphorical competence (comprehension group: Qw = 189.399, *p* = 0.000 < 0.01; general group: Qw = 129.292, p = 0.000 < 0.01; production group: Qw = 107.400, *p* = 0.000 < 0.01), which means other moderating variables could influence the effect within the subgroup.

**Table 4:** Moderator analysis of metaphorical competence type.

**Groups**	**Effect size and 95% confidence interval**						**Test of null(2-Tail)**		**Heterogeneity**			
**Group**	**Number** **studies**	**Point** **estimate**	**Standard** **error**	**Variance**	**Lower** **limit**	**Upper** **limit**	**Z-value**	***P*-value**	**Q-value**	**Df (Q)**	***P*-value**	**I-squared**
**Fixed effect analysis**												
comprehension	9	0.803	0.089	0.008	0.629	0.976	9.063	0.000	189.399	8	0.000	95.776
general	7	1.348	0.086	0.007	1.180	1.516	15.721	0.000	129.292	6	0.000	95.359
identification	12	0.423	0.070	0.005	0.286	0.561	6.046	0.000	18.439	11	0.072	40.345
Interpretation	7	0.276	0.090	0.008	0.100	0.451	3.076	0.002	9.808	6	0.133	38.824
production	16	0.882	0.064	0.004	0.756	1.008	13.746	0.000	107.400	15	0.000	86.033
Total within									454.338	46	0.000	
Total between									102.948	4	0.000	
Overall	51	0.743	0.035	0.001	0.676	0.811	21.478	0.000	557.286	50	0.000	91.028
**Mixed effects analysis**												
Comprehension	9	0.762	0.433	0.187	0.087	1.610	1.76	0.078				
General	7	1.425	0.415	0.172	0.612	2.238	3.436	0.001				
Identification	12	0.448	0.093	0.009	0.266	0.63	4.831	0.000				
Interpretation	7	0.295	0.116	0.013	0.069	0.522	2.556	0.011				
Production	16	1.286	0.2	0.04	0.893	1.679	6.419	0.000				
Total between									24.070	4	0.000	
Overall	51	0.522	0.066	0.004	0.392	0.652	7.869	0.000				

Most of the methods to test metaphorical ability are to use comprehensive test papers; there are mainly two methods for measuring metaphorical production: (a) calculating the metaphor density in subjects' expressions and (b) evaluating based on a more comprehensive test paper. A subgroup test can be further checked. Results show that the measurement method is a statistically significant moderator of the effect (Q_B_ = 10.515, *P* = 0.001 < 0.01). In 16 studies related to metaphorical production, seven studies using metaphor density as an indicator show a higher effect (d = 2.193) than nine studies using test papers (d = 0.805). The two subgroups have heterogeneity, suggesting the difference in effect may be caused by other moderators. Specific results are shown in Table 5.

**Table 5:** Moderator analysis of measuring method.

**Groups**	**Effect size and 95% confidence interval**						**Test of null (2-Tail)**		**Heterogeneity**			
**Group**	**Number** **studies**	**Point** **estimate**	**Standard** **error**	**Variance**	**Lower** **Limit**	**Upper** **limit**	**Z-value**	***P*-value**	**Q-value**	**Df (Q)**	***P*-value**	**I-squared**
**Fixed effect analysis**												
Metaphor	7	1.567	0.150	0.023	1.272	1.861	10.432	0.000	25.785	6	0.000	76.707
test paper	9	0.729	0.071	0.005	0.590	0.868	10.274	0.000	56.201	8	0.000	85.765
Total within									81.959	14	0.000	
Total between									25.441	1	0.000	
Overall	16	0.882	0.064	0.004	0.756	1.008		0.000	107.400	15	0.000	86.033
**Mixed effects analysis**												
Metaphor	7	2.193	0.370	0.137	1.468	2.918	5.929	0.000				
Test paper	9	0.805	0.216	0.046	0.382	1.227	3.734	0.000				
Total between									10.515	1	0.001	
Overall	16	1.157	0.186	0.035	0.792	1.522	6.212	0.000				

### Effects of nation/region

As shown in Table 6, regarding region factors, the average effect of 37 domestic studies was d = 0.644, and that of 14 international studies was larger with d = 1.670. The effect of explicit instruction on L2 metaphorical competence has a medium impact in China but increases significantly in global regions. The table shows Q_B_ = 8.508, *p* = 0.004 < 0.01, which means that the moderating variables' region influences the effect. A further evaluation indicates that there is significant heterogeneity in the effects of both China (Qw = 175.530, *p* < 0.01) and international groups (Qw = 310.182, *p* < 0.01). Other moderators also influence the difference in effect for both groups.

**Table 6:** Moderator analysis of nation/region.

**Groups**	**Effect size and 95% confidence interval**						**Test of null (2-Tail)**		**Heterogeneity**				**Tau-squared**		
**Group**	**Number** **studies**	**Point** **estimate**	**Standard** **error**	**Variance**	**Lower** **limit**	**Upper** **limit**	**Z-value**	***P*-value**	**Q-value**	**Df (Q)**	***P*-value**	**I-squared**	**Tau** **squared**	**Standard** **error**	**Variance**
**Fixed effect analysis**															
China	37	0.547	0.042	0.002	0.465	0.628	13.103	0.000	175.530	36	0.000	79.491	0.251	0.079	0.006
International	14	1.179	0.062	0.004	1.057	1.300	19.005	0.000	310.182	13	0.000	95.809	1.400	0.818	0.669
Total within									485.712	59	0.000				
Total between									71.574	1	0.000				
Overall	51	0.743	0.035	0.001	0.676	0.811	21.478	0.000	557.286	50	0.000	91.028	0.629	0.170	0.029
**Mixed effects analysis**															
China	37	0.644	0.094	0.009	0.46	0.828	6.864	0.000							
International	14	1.67	0.339	0.115	1.005	2.334	4.926	0.000							
Total between									8.508	1	0.004				
Overall	51	0.717	0.09	0.008	0.540	0.894	7.929	0.000							

### Effect of experimental design

From the perspective of experimental design, most researchers choose to set up experimental and control groups in their studies. By implementing the teaching intervention in the experimental group and comparing learners' scores in the post-tests, the effect of instruction on L2 metaphorical competence can be evaluated. Some researchers have no control group but choose to compare the performance of the same group in pre- and post-tests. The analysis is conducted according to the two types of experimental designs (with and without control groups).

Results in Table 7 indicate that the presence or absence of a control group does not generate statistically significant effects on the outcomes (Q_B_ = 0.146, *p* = 0.702 > 0.05).

**Table 7:** Moderator analysis of experimental design.

**Groups**	**Effect size and 95% confidence interval**						**Test of null (2-Tail)**		**Heterogeneity**			
**Group**	**Number** **studies**	**Point** **estimate**	**Standard** **error**	**Variance**	**Lower** **Limit**	**Upper** **limit**	**Z-value**	***P*-value**	**Q-value**	**Df (Q)**	***P*-value**	**I-squared**
EG-CG	38	0.852	0.039	0.002	0.775	0.628	21.619	0.000	456.414	37	0.000	91.893
No control	13	0.377	0.072	0.005	0.236	1.300	5.219	0.000	67.582	12	0.000	82.244
Total within									523.997	49	0.000	
Total between									33.289	1	0.000	
Overall	51	0.743	0.035	0.001	0.676	0.811	21.478	0.000	557.286	50	0.000	91.028
**Mixed effects analysis**												
EG-CG	37	0.644	0.094	0.009	0.46	0.828	6.197	0.000				
No control	14	1.67	0.339	0.115	1.005	2.334	4.079	0.000				
Total between									0.146	1	0.702	
Overall	51	0.844	0.114	0.013	0.621	1.068	7.409	0.000				

Further tests for heterogeneity in the effect sizes under the two experimental designs were carried out separately. The results all showed significant heterogeneity (with the control group: Q_W_ = 456.414, *P* = 0.000 < 0.01; without the control group: Q_W_ = 65.582, *P* = 0.000 < 0.05). This implies that there are still differences between effect sizes within subgroups of the two experimental designs, possibly influenced by other moderating factors.

### Effect of literature source

Regarding literature sources, there were 16 studies from dissertations and 35 journal articles in the collected literature. Table 8 indicates the mean effect size for journal articles was d = 1.052. In contrast, the mean effect size for the academic thesis was d = 0.593, which shows a difference between the mean effect sizes in academic thesis and journal articles. It can also be seen that on the variable of article source, *p* = 0.016 < 0.05, the article source has a significant effect on the metaphorical competence intervention.

**Table 8:** Moderator analysis of literature source.

**Groups**	**Effect size and 95% confidence interval**						**Test of null (2-Tail)**		**Heterogeneity**			
**Group**	**Number** **studies**	**Point** **estimate**	**Standard** **error**	**Variance**	**Lower** **Limit**	**Upper** **limit**	**Z-value**	***P*-value**	**Q-value**	**Df (Q)**	***P*-value**	**I-squared**
**Fixed effect analysis**												
Journal Paper	35	0.808	0.041	0.002	0.728	0.888	19.811	0.000	519.279	34	0.000	93.452
Thesis	16	0.577	0.065	0.004	0.449	0.706	8.818	0.000	29.077	15	0.016	48.412
Total within									548.356	49	0.003	
Total between									8.931	1	0.000	
Overall	51	0.743	0.035	0.001	0.676	0.811	21.478	0.000	557.286	50	0.000	91.028
**Mixed effects analysis**												
Journal Paper	37	1.052	0.167	0.028	0.724	1.379	6.297	0.000				
Thesis	14	0.593	0.091	0.008	0.414	0.772	6.49	0.000				
Total between									5.806	1	0.016	
Overall	51	0.699	0.08	0.006	0.541	0.856	8.716	0.000				

### Meta-regression analysis

In terms of the continuous variable, this study conducted a meta-regression test to explore the intervention length's effect on improving L2 metaphorical competence. A total of 41 individual studies reported the size of teaching interventions ranging from 2 to 24 weeks, with the average length being 9.12 weeks. Results showed that while intervention length positively impacts improving L2 metaphorical competence, the impact could be more considerable. The regression equation in Table 9 is Y = 0.0481X+0.2521. This means that when the intervention length increases, the effect of the intervention on L2 metaphorical competence will become more significant to some degree.

**Table 9:** Regression results of intervention length and intervention effect.

**Main results for model 1, random effect (ML), Z-distribution, Std diff in mean**						
**Covariate**	**Coefficient**	**Standard error**	**95% Lower**	**95% Upper**	**Z-value**	**2-Sided *p*-value**
Intercept	0.2521	0.2545	−0.2466	0.7508	0.99	0.3218
Lenght	0.0481	0.0194	0.0100	0.0862	2.47	0.0134

Statistics for model 1.

Test of the model: simultaneous test that all coefficients (excluding intercept) are zero.

Q = 6.12, df = 1, p = 0.0134.

Goodness of fit: test that unexplained variance is zero.

Tau^2^ = 0.5659, Tau = 0.7375, I^2^ = 90.72%, Q = 452.82, p = 0.0000.

The meta-regression results showed that the effect of a short intervention was not significant, further validating that the improvement of metaphorical competence is considered a long-term process.

**Figure 4:**
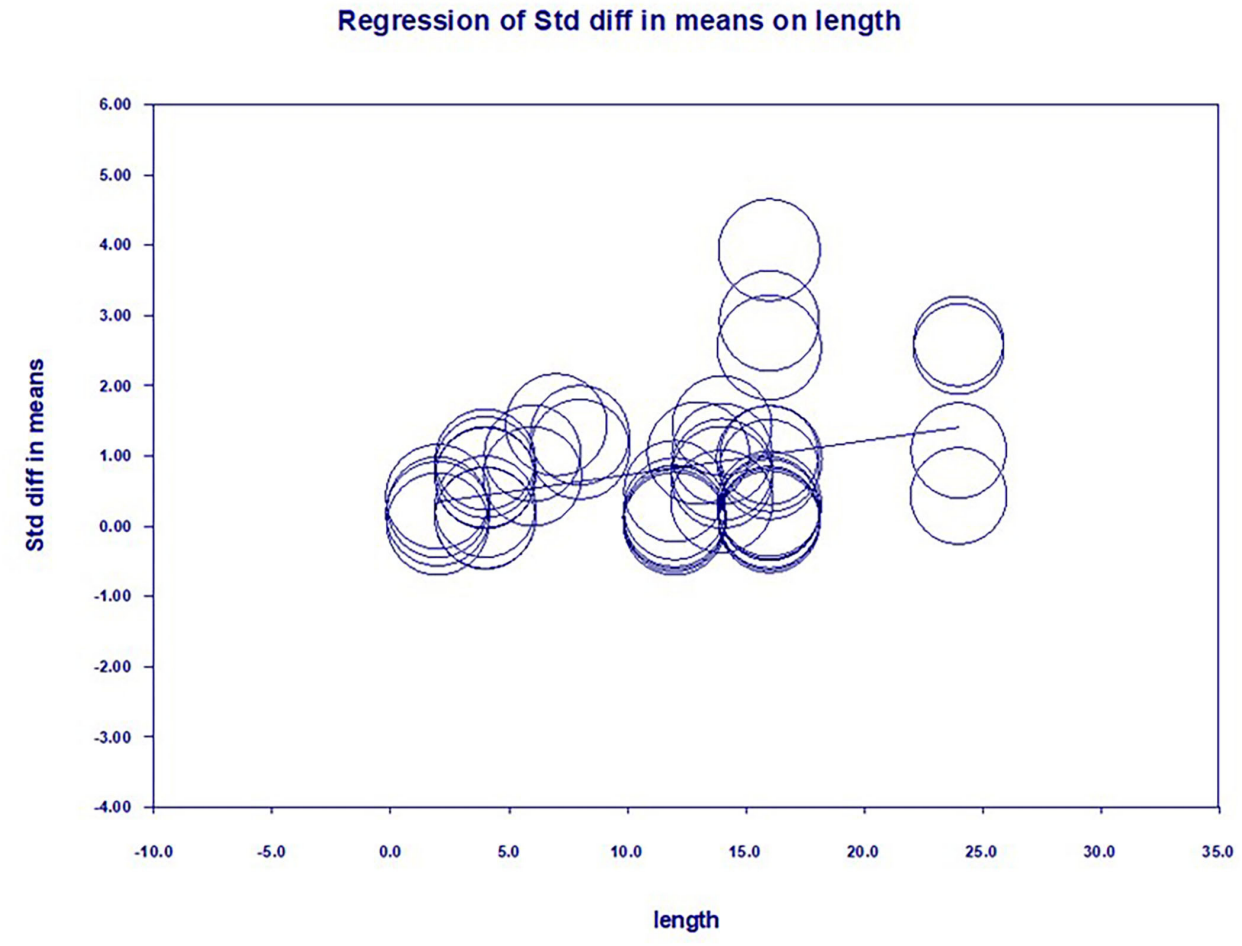
Regression curve of intervention length and intervention effect.

Based on the Figure 4, literature included in the present meta-analysis and the best effect was achieved when the length of the intervention was around 16 weeks, which is consistent with the study design of most studies. Results were significant (Q = 452.82, df = 42, *p* = 0.0000 < 0.01), suggesting that the moderating variable intervention length did not explain all of the variances and that there may be other moderating variables.

## Discussion

A widely accepted idea is that metaphorical competence is teachable. Researchers have attempted to verify the possibilities of L2 metaphorical competence cultivation from both theoretical and empirical perspectives (Boers and Littlemore, 2000; Yasuda, 2010). Teaching activities were based on the idea that they made learning idioms simpler and boosted retention; they learned to infer the meanings of new orientation idioms from their settings and utilize them more effortlessly (Karatay et al., 2022; Younas et al., 2022a). Most instructional interventions for L2 metaphorical competence follow a similar path. This study tried to analyze the metaphors used by language students who preferred the situational learning style to their current cognitive learning (Pishghadam et al., 2009). Firstly, learners were introduced to the concept of metaphor and related knowledge of metaphor to increase their metaphorical awareness, and then they were given exercises to improve their metaphorical competence. The main effect was tested to draw a general conclusion on the impact of metaphorical competence interventions. The findings showed that the overall development of the 51 individual studies reached a considerable level (d = 0.88), and the 95% confidence interval was [0.654, 1.123], which indicated that the existing metaphorical competence interventions indeed achieved a significant effect. It was consistent with the conventional view that interventions for L2 metaphorical competence are effective. Liu et al. (2020) utilized a modified Stroop paradigm to study how Chinese culture metaphorically translates abstract moral notions into tangible referents. Chen et al. (2018) also revealed that the source domain's relationship to the destination domain affects the development of metaphor. The study employed a variant of the Stroop paradigm to investigate how the Chinese language is used to provide concrete metaphors for abstract moral principles (Liu et al., 2020). In the by Shirazi and Talebinezhad (2013), the subjects discussed a topic in the pretest. They were asked to have another discussion on the same issue after the instructional intervention. As the subjects already knew the content of the test well, the difficulty of the two trials was different for them.

In contrast to nominal metaphors, which employ figurative language based on nouns, predicate metaphors involve the metaphorical abstraction of concrete verbs, which often include more significant action and motor simulation than nouns. An analysis of metaphorical competence indicated that metaphorical teaching interventions reflected different effects on metaphorical ability with different focuses (Q_B_ = 24.070, df = 4, *P* = 0.000 < 0.01). A large-level effect could be seen in improving metaphorical comprehension (d = 0.762), production (d = 1.286), and general metaphorical competence (d = 1.425). The average effect on metaphorical identification was at a medium level (d = 0.448). In comparison, the improvement of metaphorical interpretation was the least effective (d = 0.295). It may be strongly related to the interference of native language concepts. Zhai et al. (2018) clarified how cognition and emotion process vertical and spatial metaphors of moral concern. Following advancements in language teaching and learning, metaphor analysis is used indirectly to analyze university students' views on learning and teaching (Pishghadam and Pourali, 2011). These two equally impact the metaphorical relationship between morality and verticality, but emotion is procured quickly. Verbal metaphors are processed *via* simulation and abstraction, and their meaning is linked with the phrase's meaning (Li et al., 2022). Learners often rely on their native language, using the familiar concepts in the native language system to project the corresponding categories in the second language learning (Feng and Zhou, 2021; Noor et al., 2022).

Both sign and spoken languages use metaphors. Sign languages use metaphors differently than spoken languages. In this study, we compared sign and spoken metaphors (Meir and Cohen, 2018). When dealing with metaphorical expressions, they may activate their metaphorical knowledge system, facilitating their comprehension and production (Chen and Lai, 2015). The role of intervention is evident.

Nevertheless, in metaphor interpretation, subjects depend more on their cognitive abilities and should put in more effort to understand unfamiliar concepts. A regression test was conducted to examine the effect of the intervention length on the development of L2 metaphorical competence. A total of 41 studies reported the size of teaching interventions ranging from 2 to 24 weeks, with the average length being around 9 weeks. The meta-regression results showed a positive relationship (*P* = 0.0134 < 0.05). According to the regression equation Y = 0.0481X+0.2521, it could be seen that for each unit increase in intervention length, the corresponding effect of the intervention increased by an average of 0.0481 units. However, the degree of variation was relatively small. The linear regression table for the intervention period showed that the best effect sizes for the L2 metaphorical intervention were found at around 16 weeks, which was in line with the intervention design of most studies.

A view argues that it is almost impossible to understand and use foreign concepts in the same way as native speakers of a foreign language; several studies have proved that the development of metaphorical skills is a gradual process and can be developed through conscious instruction and practice. Not only did the intervention have an effect immediately, but it also positively affected the learner's future language learning (Yang and Peng, 2021; Younas et al., 2022b). The previous study explains that conflict intensity modifies the temporal trajectory of metaphor processing in Mandarin, and animacy violation may assist the integration of the reanalysis stage for metaphorical understanding (Ji et al., 2020). Shen et al. (2015) examine if mental imaging ability impacts sensory-motor participation during action metaphor understanding. The results showed that the students realized that the root systematic metaphor method was helpful for vocabulary acquisition. After the intervention, their attitude toward the critical role of metaphor learning changed positively, and they showed a strong desire for metaphor learning. Appropriate metaphorical teaching does not add an extra burden to learners but rather positively impacts them. From this perspective, metaphorical instruction should be given enough attention. Compared with traditional language teaching, metaphorical instruction places greater demands on teachers. They have a long way to go in improving learners' L2 metaphorical competence to achieve the higher goal of second language education. In the future, more research on the effectiveness of metaphorical teaching interventions is needed to find better ways to use metaphors to teach Chinese as a second language.

## Conclusion

This meta-analysis showed that the current instructional intervention on L2 metaphorical competence had a significant effect. It indicated that explicit teaching methods were effective in raising learners' metaphorical awareness and could improve their L2 metaphorical competence to a certain extent. Subgroup analyses were conducted to further explore the moderating factors' effects, and results indicated that direct teaching interventions effectively improved different aspects of metaphorical competence. A significant effect could be found in the abilities of metaphorical comprehension, production, and general metaphorical competence. The least effect of instructional interventions was demonstrated in metaphorical interpretation skills.

Regarding the two methods of measuring metaphorical production, the metaphor density task could generate a higher impact than the sentence task. The experimental design did not have statistically significant effects on the results. Both these two kinds of experimental designs have shown a relatively large effect. The region had a significant moderating effect on the L2 metaphorical competence intervention. The impact of metaphorical competence intervention in international nations was significantly better than in China. Using a metaphor is one thing, but using an emotionally positive metaphor is something else. Since, in this study, the emo-sensory nature of language was not taken into account, another study can be done to measure it (Akbari and Pishghadam, 2022; Pishghadam et al., 2022). The literature source was an essential moderating factor in the size of the effect of the metaphorical competence intervention. The meta-regression showed that the length of the intervention had a positive effect on the effect of improving metaphorical competence.

## Data availability statement

The raw data supporting the conclusions of this article will be made available by the authors, without undue reservation.

## Ethics statement

The studies involving human participants were reviewed and approved by School of Foreign Languages, East China Normal University. The patients/participants provided their written informed consent to participate in this study.

## Author contributions

XZ collected data and wrote the article. MY collected the data and guided the psychological perspective and methodology. LG analyzed the data. LG and AO contributed to the discussion section. AO helped with major revisions and proofread and finalized the manuscript. All authors contributed to the article and approved the submitted version.

## Conflict of interest

The authors declare that the research was conducted in the absence of any commercial or financial relationships that could be construed as a potential conflict of interest.

## Publisher's note

All claims expressed in this article are solely those of the authors and do not necessarily represent those of their affiliated organizations, or those of the publisher, the editors and the reviewers. Any product that may be evaluated in this article, or claim that may be made by its manufacturer, is not guaranteed or endorsed by the publisher.
